# How anisotropic and isotropic atomic displacement parameters monitor protein covalent bonds rigidity: isotropic B-factors underestimate bond rigidity

**DOI:** 10.1007/s00726-021-02985-x

**Published:** 2021-04-29

**Authors:** Oliviero Carugo

**Affiliations:** 1 Department of Chemistry, University of Pavia, Viale Taramelli 12, 27100 Pavia, Italy; 2 Department of Structural and Computational Biology, University of Vienna, Vienna, Austria

**Keywords:** Atomic displacement parameter, Atomic resolution, B-factor, Covalent bond, Hirshfeld rigidity test, Protein structure

## Abstract

Under the assumption that covalent bonds are rigid, it is possible to compare the estimations of rigidity based on anisotropic and isotropic B-factors. This is done by computing the difference of the mean-square displacements (Delta-u) of atoms A and Z along the covalent bond A–Z, which must be close to zero for a rigid bond. The analysis of a high-quality set of protein structures, refined at a resolution better than (or equal to) 0.8 Angstroms, showed that Delta-u is significantly close to zero when anisotropic B-factors are used, with an average 60% Delta-u reduction. This reduction is larger for larger B-factors and this suggests that care should be taken in data-mining procedures that involve isotropic B-factors, especially at lower resolution, when anisotropic B-factors cannot be determined and when the average B-factor increases.

**Supplementary Information:**

The online version contains supplementary material available at 10.1007/s00726-021-02985-x.

The benefits of high-resolution macromolecular crystal structures have been repeatedly described (Longhi et al. 1998; Dauter et al. 1997; Schmidt and Lamzin 2002; Schmidt and Lamzin 2010). The wealth of details in electron density maps at high resolution allows the characterization of the conformational disorder of many protein atoms, which may show two or three stable positions, and of the protein hydration by water molecules that cover the protein surface (Schmidt and Lamzin 2010; Schmidt et al. 2011; Bhattacharjee and Biswas 2011). A remarkable feature of high-resolution crystal structures is the anisotropic refinement of the atomic displacement parameters (therein after referred to as B-factors), which is impossible at lower resolution and which provides valuable information on local dynamics (Schmidt et al. 2011; Carugo 2020; Carugo 2019a). However, a systematic comparison—based on independent and external criteria—of anisotropic B-factors, which can be refined only at high resolution, and isotropic B-factors, which are routinely refined at lower resolution, have never been published.

Anisotropic and isotropic B-factors can be compared by analyzing their ability to monitor the rigidity of the protein covalent bonds, a feature that is independent of the refinement level but is directly related to the electronic structure of the proteins.

Covalent bonds are highly rigid and their deformation implies severe energy costs (Slater 1968). This has been exploited in macromolecular crystallography with the introduction of refinement restraints (Tronrud 1996; Thorn et al. 2012; Parois et al. 2018), which essentially assume that the difference of the mean-square displacements of atoms A and Z along the covalent bond A–Z must be close to zero. These restraints are usually relaxed at very high resolution, when they are no more essential to ensure a physically and chemically realistic structure description.

Axiomatically, it is possible to assume that covalent bond is rigid, especially at low temperature—crystal structures are routinely determined at 100 K nowadays—and this is monitored by the Hirschfeld test (Hirshfeld 1976), according to which, as mentioned above, the components of the B-factors of the two atoms along the covalent bond must be the same.

When isotropic B-factors (B) are available, the mean-square displacement (u) of an atom is the same in all directions around the atomic average position, and the rigidity of the bond A–Z can be monitored by the function *Delta-u*, defined as:

1 $$ {\text{Delta}} - u = \left| {u_{A} - u_{Z} } \right| = \left| {\sqrt {\frac{{B_{A} }}{{8\pi^{2} }}} - \sqrt {\frac{{B_{Z} }}{{8\pi^{2} }}} } \right|, $$

which must be close to 0 Å, according to the Hirshfeld rigidity test (Hirshfeld 1976) (*B*_*A*_ and *B*_*Z*_ are the B-factors of atoms A and Z). With anisotropic B-factors (**U**), the *Delta-u* function must consider that the mean-square displacement (u) of an atom is not the same in all directions, and it must be computed as:

2 $$ {\text{Delta}} - u = \left| {n^{{\text{T}}} {\text{U}}_{{\text{A}}} n - n^{{\text{T}}} {\text{U}}_{{\text{Z}}} n} \right|, $$

where *n* is the unit vector in the covalent bond direction, and ***U***_***A***_ and ***U***_***Z***_ are the anisotropic B-factors of atoms A and Z (Burgi 1994).

In the present communication, isotropic and anisotropic *Delta-u* values were computed and compared in a non-redundant set of extremely high-resolution protein crystal structures extracted from the Protein Data Bank (Bernstein et al. 1977; Berman et al. 2000).

Only X-ray crystal structures refined at a resolution of at least 0.8 Å and determined in the 90–100 K temperature range were retained, and the following thirty crystal structures were eventually kept—chain identifiers in parentheses—once the sequence redundancy was reduced to 40% pairwise sequence identity with CD-HIT (Li and Godzik 2006; Fu et al. 2012): 1ejg(A), 1gci(A), 1iua(A), 1r6j(A), 1ucs(A), 1us0(A), 1w0n(A), 1 × 6z(A), 2b97(A), 2ixt(A), 2izq(A), 2ov0(A), 2pve(A), 2vb1(A), 2wfi(A), 3mfj(A), 3ui4(A), 3 × 2 m(A), 3 × 34(A), 4hp2(A), 4rek(A), 4ua6(A), 5al6(A), 5kwm(A), 5nfm(A), 5tda(A), 5yce(A), 6e6o(A), 6l27(A), 6s2m(A).

Solvent accessible surface areas for each atom were computed with Naccess (Hubbard and Thornton 1993) and all the other computations were performed with locally written software.

Anisotropic *Delta-u* was computed for all protein covalent bonds with anisotropic B-factors (Eq. 1), and isotropic *Delta-u* was computed with the equivalent isotropic B-factors (Eq. 2), which, for anisotropically refined atoms, are equal to

3 $$ B = 8\pi^{2} \frac{{U_{11} + U_{22} + U_{33} }}{3}. $$

In general, and as expected, anisotropic *Delta-u* is smaller than isotropic *Delta-u* for all types of covalent bonds (see an example in Figure S1, Supplementary Material): on average, it is 0.0108 (0.0001) Å smaller. This difference is larger for side-chain bonds [0.0157 (0.0002) Å] than for main-chain bond [0.0061 (0.0002) Å]. Only for 13% of the bonds, the isotropic *Delta-u* is slightly smaller than the anisotropic *Delta-u* and most of these cases concern the C–N backbone bonds. This percentage is smaller for side-chain bonds (10%) than for backbone bonds (17%).

This is certainly not surprising. In fact, given the assumption that covalent bonds are rigid, this reflects the better modeling of atomic dispersion around the equilibrium positions in anisotropic refinements. In other words, the rigidity of covalent bonds is better accounted for by anisotropic B-factors refinements, especially for side-chains, which tend to be more flexible.

However, the added value of the work presented here is the fact that this is a quantitative comparison, which points out that, given that the anisotropic and isotropic *Delta-u* are equal, on average, to 0.0075 (0.0001) and 0.0184 (0.0001) Å, the deviation from rigidity of the covalent bonds is reduced by 60% when anisotropic B-factors are refined. This improvement is remarkable and somehow surprising by its amount.

This can be appreciated also by the relationship between the anisotropic and isotropic *Delta-u* shown in Fig. 1a. While the isotropic *Delta-u* increases considerably, from values close to 0 Å up to 0.04 Å, the anisotropic *Delta-u* increases much less, only from 0.006 to 0.007 Å to about 0.01 Å. This confirms that the covalent bond rigidity is drastically better monitored by anisotropic B-factors.

**Fig. 1:**
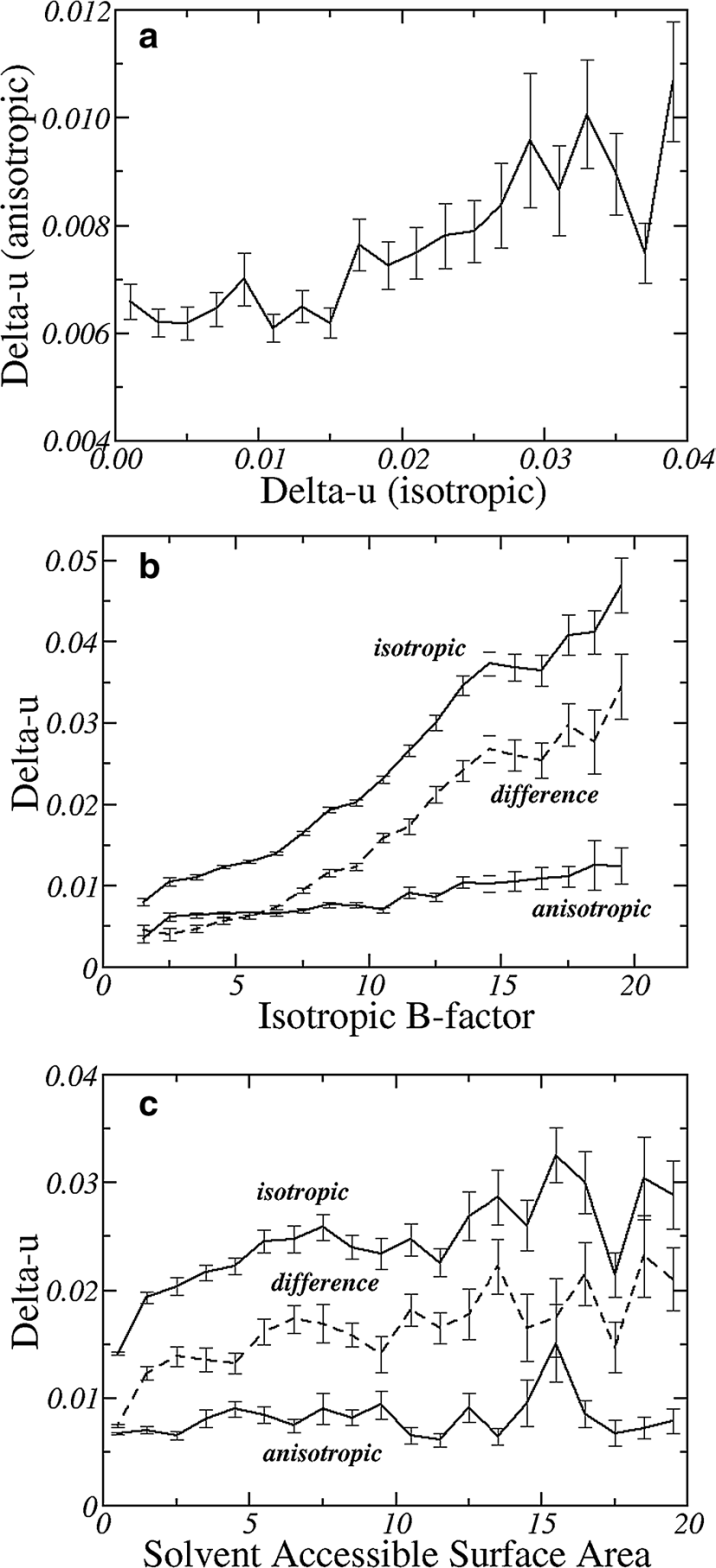
Relationship between isotropic and anisotropic Delta-u (Å, **a**); between the Delta-u (Å) and the average isotropic B-factor of the two atoms that are covalently bound (Å^2^; **b**); and between the Delta-u (Å) and the average solvent accessible surface area of the two atoms that are covalently bound (Å^2^; **c**). Both backbone and side-chain bonds, which are equally numerous, were considered

Interestingly, both anisotropic and isotropic *Delta-u* tend to be larger for atom pairs that have a larger average isotropic B-factor (Fig. 1b). However, this is more pronounced for isotropic *Delta-u*, which increases from 0.01 to 0.04 Å when B increases from 5 to 20 Å^2^—note that the latter value is close to the maximum possible B value at very high resolution (Carugo 2018a, 2019b). On the contrary, the anisotropic *Delta-u* increases much less in the same B-factor range. This is represented, in the figure, by the fact that also the difference between isotropic and anisotropic *Delta-u* increases if the B-factors increase. This clearly indicates that large atomic positional dispersions cannot be described effectively by isotropic B-factor.

Similar trends are observed when the average solvent accessible surface area of the atoms that are covalently bound is considered (Fig. 1c). The isotropic *Delta-u* values increase if the solvent accessible area surface area increases, indicating that the rigidity is larger for covalent bonds buried in the protein core than for covalent bonds exposed to the solvent. On the contrary, the anisotropic *Delta-u* is nearly constant in the examined range of solvent accessible surface area and, as a consequence, the difference between isotropic and anisotropic *Delta-u* increases if the solvent accessibility increases. This supports the previous observations on the relationships between *Delta-u* and equivalent isotropic B-factor and is not surprising since larger isotropic B-factors are expected for atoms more exposed to the solvent.

The data available in the Protein Data Bank allow one to estimate that the covalent bond rigidity is much better accounted for by anisotropic B-factors than by isotropic B-factors. A remarkable 60% reduction of the deviation from rigidity is observed, on average. If on the one side this is expected, on the other side, it points out that the information provided by isotropic B-factors is of limited accuracy when protein dynamics must be quantitatively evaluated at a molecular level. Care should then be taken in data-mining procedures that involve isotropic B-factors (Carugo 2018b; Sun, Qu, Feng, Reetz 2019), from drug design (Johnson et al. 2018), to atom position accuracy estimation (Dinesh Kumar et al. 2015), or to protein engineering (Reetz et al. 2006).

It is also important to remember that the inaccuracies of isotropic B-factors estimated in the present communication might be underestimated, since isotropic *Delta-u* was computed with isotropic equivalent B-factors and not with genuine isotropic B-factors. In other words, isotropic *Delta-u* was computed with isotropic B-factors that resulted from anisotropic refinements and not with isotropic B-factors that can be refined at lower resolution.

## Supplementary Information

Below is the link to the electronic supplementary material.

Supplementary file1 (DOCX 149 KB)
