# Supplementary material for: How anisotropic and isotropic atomic displacement parameters monitor protein covalent bonds rigidity: isotropic B-factors underestimate bond rigidity
Source: Amino Acids. 2021 Apr 29;53(5):779–82. doi: 10.1007/s00726-021-02985-x (PMC8128831; doi:10.1007/s00726-021-02985-x)
Supplement: Supplementary file 1 — Supplementary file1 (DOCX 149 KB) [file 726_2021_2985_MOESM1_ESM.docx]

How anisotropic and isotropic atomic displacement parameters monitor protein covalent bonds rigidity – Isotropic B-factors underestimate bond rigidity

Oliviero Carugo

Department of Chemistry, University of Pavia, Pavia, Italy and Department of Structural and Computational Biology, University of Vienna, Vienna, Austria (ORCID 0000-0002-2924-9016)

Correspondence to:

Oliviero Carugo

Department of Chemistry

University of Pavia

Viale Taramelli 12

I-27100 Pavia, Italy

Email: [Oliviero.carugo@univie.ac.at](mailto:Oliviero.carugo@univie.ac.at)

# Supplementary Material

|  |
| --- |
| **Figure S1**. View of glycine 20 of chain A in the PDB file 1ejg. Thermal ellipsoids are shown with Chimera (https://www.cgl.ucsf.edu/chimera/; Tools-Structure Analysis-Thermal Ellipsoids, with scale factor = 2). For each atom, the value of the isotropic B-factor is shown in parentheses. The isotropic *Delta-u* values are considerably larger than the anisotropic counterparts for each of the three covalent bonds. This is a consequence of the considerable anisotropy of all the atoms, which is equal to 4.238 for atom N, 3.402 for atom Cα, 3.158 for atom C, and 3.678 for atom O (the anisotropy is defined as it follows: the eigenvalues λ1 ≥ λ2 ≥ λ3 of the **U** tensor are computed and the anisotropy is equal to λ1/λ3). |
